# An Introduction to the Avian Gut Microbiota and the Effects of Yeast-Based Prebiotic-Type Compounds as Potential Feed Additives

**DOI:** 10.3389/fvets.2015.00028

**Published:** 2015-09-02

**Authors:** Stephanie M. Roto, Peter M. Rubinelli, Steven C. Ricke

**Affiliations:** ^1^Department of Food Science, Center for Food Safety, University of Arkansas, Fayetteville, AR, USA

**Keywords:** poultry, microbiota, lactobacillus, *Bifidobacterium*, yeast

## Abstract

The poultry industry has been searching for a replacement for antibiotic growth promoters in poultry feed as public concerns over the use of antibiotics and the appearance of antibiotic resistance has become more intense. An ideal replacement would be feed amendments that could eliminate pathogens and disease while retaining economic value via improvements on body weight and feed conversion ratios. Establishing a healthy gut microbiota can have a positive impact on growth and development of both body weight and the immune system of poultry while reducing pathogen invasion and disease. The addition of prebiotics to poultry feed represents one such recognized way to establish a healthy gut microbiota. Prebiotics are feed additives, mainly in the form of specific types of carbohydrates that are indigestible to the host while serving as substrates to select beneficial bacteria and altering the gut microbiota. Beneficial bacteria in the ceca easily ferment commonly studied prebiotics, producing short-chain fatty acids, while pathogenic bacteria and the host are unable to digest their molecular bonds. Prebiotic-like substances are less commonly studied, but show promise in their effects on the prevention of pathogen colonization, improvements on the immune system, and host growth. Inclusion of yeast and yeast derivatives as probiotic and prebiotic-like substances, respectively, in animal feed has demonstrated positive associations with growth performance and modification of gut morphology. This review will aim to link together how such prebiotics and prebiotic-like substances function to influence the native and beneficial microorganisms that result in a diverse and well-developed gut microbiota.

## Introduction

Poultry production in the past century has transitioned from predominantly breeding layers to breeding a mixture of both layers and broilers, based on the evolution of consumer demand (1–3). Success in the optimization of different broiler lines is due to genetics as well as optimizing diets with more precise nutritional formulations (4, 5). Comparison of individual genetic lines has revealed differing intestinal development, feed intake, and digestibility traits among other characteristics, which may impact performance (6–9). Improved diets have allowed broilers to reach their optimum body weight and feed conversion rate while minimizing mortality. Comparing poultry diets from the 1950s to those of the 1990s and 2000s illustrates the progress made (10, 11). For example, broiler chickens raised on a typical diet in 1957 had an average weight of 1,430 g at 84 days of age, whereas broilers fed a diet from 2001 yielded an average weight of 5,520 g at the same age. The feed conversion ratio in 2001 (2.68) was also considerably better compared to 1957 (3.26) (11). The current poultry diet contains the appropriate balance of amino acids, fatty acids, major and trace minerals, energy, and protein necessary for optimum growth (12).

Supplementation of various biologics have been attempted to enhance poultry feed for maximum growth development and health. Antibiotics enhance growth and reduce pathogens and although the exact mechanisms remain unclear, numerous working hypotheses have been offered (13–17). Antibiotic incorporation into poultry feed has since been tightly restricted and/or omitted due to microbial antibiotic resistance, presumably originating from both poultry (among other livestock) and humans (18–20). Since the exclusion of antibiotics in diets, a number of alternative supplements have been tried (Table 1), including prebiotics (21).

**Table 1:** **Commonly researched feed additives for host health, including growth promotion and pathogen prevention, used in animal feed, their modes of action, and reviews for references**.

Compound	What they do	How they work	Reviews for reference
Prebiotic	Food ingredient to act as substrate for beneficial bacteria in the host GIT microbiota	Host consumes prebiotic and it endures through the GIT relatively intact to the lower intestines where it selectively acts as substrate for beneficial bacteria	(22–24)
Probiotic	Live microbial feed supplements that beneficially impact intestinal microbial balance	Competes with pathogenic bacteria to colonize the intestines; ferments substrates to produce short-chain fatty acids; stimulates the immune response of the host	(23, 25, 26)
Mannan-oligosaccharide	Specific oligosaccharide that inhibits pathogenic bacteria from binding the mucosal epithelial lining	Pathogens have receptors specific for mannan residues, the pathogenic bacteria binds the mannan and does not bind to the host epithelial cells	(27–29)
Organic acid	Reduce the number of pathogens	Undissociated form traverses the bacterial cell membrane; once inside the bacterial cell, the organic acid dissociates to produce H^+^ ions, which lowers the pH. The bacterial cell then has to expend its energy to restore it natural balance rather than promote its own growth	(30–34)

A prebiotic, as defined by Gibson and Roberfroid (35), is “a non-digestible food ingredient that beneficially affects the host by selectively stimulating the growth and/or activity of one or a limited number of bacteria in the colon and thus improves health.” This definition has been subsequently refined to include the requirements for resistance to the acidic gastric environment, gastric enzymes, gastrointestinal absorption, and fermentation by the gastrointestinal microbiota while stimulating growth of beneficial intestinal bacteria (22). Being indigestible by the upper gastrointestinal tract (GIT) enables it to enter the lower GIT as a substrate for health-promoting bacteria, such as bifidobacteria and lactobacilli, thereby modulating the microbiota (35). Many feed additives currently used do not fit wholly into the strict prebiotic classification; they may lack one or more of the criteria set by Roberfroid (22). Although these substances have differing modes of action compared to prebiotics, they have a similar end result of a healthy and mature GIT microbiome. They may inhibit pathogenic invasion, reduce pathogens in the environment, modulate the host immune response, or enhance the host GIT morphology to enable the host to better limit pathogens in the GIT lumen. These substances will be referred to as prebiotic-like substances for the remainder of this review.

The objective of this review is to provide an overview of the effects of prebiotic-like substances, particularly those that are yeast-derived, while assessing the influence on microbial diversity of the poultry gut microbiota when using single or complex mixtures. In order to achieve this, both the gut microbiota as well as prebiotics is reviewed. Additionally, the characteristics of complex mixtures of prebiotic-like substances are assessed, including their effects on the gut development and physiology, the interactions that occur between host and microorganisms, and the potential use of prebiotic-like substances in creating a more healthy gut microbiota. This review includes findings from not only poultry but also human and animal models, which may provide insight into potential effects in poultry.

## Gut Microbiome: Terminology and Definitions

The microbiota is defined as the diverse population of microorganisms in a given environment, while the microbiome can be defined by either its genetic or ecological capacities (36). Genetic diversity is the entire collection of genes of the microorganisms in an environment, while the ecological diversity is all the microorganisms that make up an ecosystem (36). The term “microflora,” once commonly used, is now often replaced by “microbiota” to avoid the plant connotation from the suffix “flora.” (36). Regardless of the term used, it is essential to use a modifying adjective when referring to a specific anatomical region. For example, “gut microbiome” is indicating only the microorganisms in the GIT. There are numerous microbiome sites in addition to the gut microbiome, as they can be any shared anatomical sites between a community of microorganisms (commensal, pathogenic, or symbiotic) (37–39). An oral microbiome, for example, is the community of microorganisms that interact with and live within the oral cavity. It has several distinct microbial habitats within the oral cavity (gingival, tongue, and teeth) and extensions of the oral cavity (esophagus, middle ear, and nasal passages). Each different habitat within the oral cavity has its own distinct bacterial population in the form of complex biofilms (40). Research has shown that even the distinctive sites of the tongue – the dorsal and the lateral regions – possess differing bacterial profiles (41). Other frequently studied sites of microbiomes are the skin and the respiratory tract (42–44). The various regions and diversity among bacterial communities of the microbiota are indicative of the inherent complexity of microbiome research.

The gut microbiome is a widely studied topic because of its impact on health as well as its characteristic intricacy. The gut microbiome is home to one of the densest bacterial populations on earth, with numbers ranging from 10^8^ to 10^14^/g of digesta (45, 46). The microbiome encompasses biochemical and metabolic pathways not found in the host genome; this attests to the extent to which the microbiome has evolved (47). Microorganisms that comprise the gut microbiota have been found to directly impact the health of the host, providing protection against epithelial damage, aiding in digestion, and promoting development of a healthy immune system (48, 49). Commensal bacteria, in the GIT of animals, aid in absorption of nutrients as well as enhance nutrient utilization (50). Additionally, research conducted thus far has shown that earlier development of a mature and diversified microbiota leads to better growth and fewer health issues, such as obesity, allergies, and asthma (51, 52). This is in part due to healthy competition among microorganisms.

## Avian Gut Anatomy, Structure, and Functionality

For a thorough understanding of the microbial communities that inhabit the GIT of poultry and the effects they may have, a brief description of the poultry GI system is warranted. The GIT of poultry, chickens specifically, begins at the esophagus and continues down past the crop, proventriculus, and gizzard, through the intestines (duodenum, jejunum, ileum, and ceca), and ends at the colon and cloaca (53, 54). The gut microbiota generally refers to the intestinal regions and the studies included in this review focus on the duodenum, jejunum, ileum, ceca, and fecal contents as well as the structural characteristics to illustrate the gut microbiome of poultry. The ceca and their contents are most often studied based on their slow passage rate [comparatively, gut transit time from mouth to the lower ileum is approximately 3 h, while contents may be retained in the ceca as long as 35 h (55–57)] as it exhibits the most diversification in the bacterial communities it harbors, in turn, indicating its impact on host health (54).

The intestines are multi-layered tubes, containing epithelial, muscular, and mucosal layers (58). Each section of the intestine, from the most proximal duodenum passing through the jejunum and out to the most distal ileum, contains numerous folds and is lined with villi and crypts. The villi are finger-like projections on the surface of the mucosal lining responsible for increasing surface area to maximize nutrient absorption and containing a meshwork of capillaries to allow nutrients entry into the bloodstream (59). When moving in the distal direction from the duodenum down toward the ileum, the mucosal lining reduces in thickness. The villi length and crypt depth also decrease in a continual gradation, which supports the notion of the majority of nutrient absorption occurring in the small intestine (58). Reduced intestinal weight is associated with improved nutrient absorption (60). Microscopic analysis has revealed that the reduction of intestinal weight is due to thinning of the epithelial lining rather than to the reduction in intestinal length, which is suggested to allow for improved nutrient absorption (61, 62).

The pancreas functions in hydrolysis of macromolecules, releasing digestive enzymes into the duodenum responsible for the hydrolysis of proteins, carbohydrates, and lipids supplied by the diet. In addition to enzyme production, the pancreas also produces hormones and bicarbonate that aid in metabolism regulation and buffer the intestinal pH, respectively (59, 63). The addition of enzymes to the duodenum allows for the small intestine to be the primary site of nutrient digestion and absorption. Having a general understanding of the digestive system of poultry allows for a more thorough insight into how microorganisms may impact GIT physiology. Turk (58) provides a more encompassing review of the entire avian GIT.

## Avian Gut Microbiome Characterization

Characterization of microbial communities native to the poultry GIT began in 1901 and has since revealed these communities to be both diverse and dynamic (64). As biased culture-based methods advanced to molecular and sequencing techniques, a broader, more comprehensive representation of the microbiome has been recognized (64, 65). Researchers have attempted to determine a bacteriological profile of the poultry GIT via 16S rRNA gene-based studies; the findings have demonstrated that the majority of the 16S rRNA sequences in the cecal contents are not-yet-identified bacterial species (64, 66, 67). These discoveries uncovered the shortcomings of previously employed culture-based methods. For example, comparison of results obtained from Zhu et al. (64) and Rada et al. (68) found differing levels of *Bifidobacteria*-species present in untreated chicken cecal contents. Zhu et al. (64) used temporal temperature gradient gel electrophoresis followed by sequencing of the 16S rRNA fragments, while Rada et al. (68) used selective media; the experimental designs of both were comparable. The works of Zhu et al. (64) and Rada et al. (68) are two such examples for the characterization of the GIT microbiome; various techniques have been attempted to ascertain the microbial populations present in the different regions of the intestinal tract (Table 2).

**Table 2:** **Research conducted on commensal bacteria in poultry GIT based on location**.

Host	Site(s)	Age(s)	Commensal or pathogenic	Method of investigation	Reference
Chicken	Ileum, cecum	7, 13 days	Commensal	PCR-based DGGE; 16S rRNA gene library analysis; qPCR	(69)
Chicken	Ileum	4, 8, 14, 21, 35 days	Commensal	DGGE; RFLP	(6)
Chicken	Ileum, cecum	4 weeks	Commensal	Percent G + C profiling	(70)
Chicken	Cecum, intestines	4, 14, 25 days	Pathogenic	Primers (species-specific) of 16S rDNA	(71)
Chicken	Cecum	1 day, 1, 2, 4, 6 weeks	Commensal	TTGE; 16S rRNA gene sequencing	(64)
Chicken	Crop, ileum, cecum, rectum	40, 41 days^a^	Commensal	16S rDNA sequencing	(72)
Chicken	Ileum, cecum	28 days	Commensal	FISH with 16S rRNA oligonucleotides	(73)
Chicken	Crop, duodenum, colon	2 months	Commensal	FCM-FISH	(74)

*^a^Indicates differing rearing methods: conventionally raised and organically raised, respectively*.

Each area of the intestinal tract harbors distinct microbial communities. For example, the cecal contents exhibit greater levels of *Clostridiaceae*-related sequences as opposed to the ileum where more abundance of *Lactobacillus*-related sequences occurs (75). Apajalhti et al. (70) used G + C content to demonstrate similar results: the measurement of bacterial communities present in the ceca and ileum exhibited considerable variation when comparing the two G + C profiles. Variation in microbial communities is not only limited to differing organs, there is also a temporal factor in the nature of the microbiome (76). The cecal contents of younger birds appeared to possess more transient communities that matured into communities with much greater complexity, while the ileum indicated an overall constant microbiome except at days 3 and 49 (the youngest and oldest sampling points) (75). The response to newly introduced microorganisms also appears to be dependent on sex of the host when analyzed in a mouse model; male and female GIT microbiota influence the metabolic activities and immune system differently (77). The concept of host factors affecting microbial diversity offers the opportunity to use established and healthy microbiomes to generate a working GIT microbial profile. However, this may prove to be quite challenging as it has been found that chickens interacting together in the same conditions, receiving the same feed, and of the same age and sex still display uniquely dominant bacterial communities (78). Although the exact quantities and qualities of a healthy microbiota have yet to be determined, a relationship appears to exist between the establishment of a mature intestinal microbiome and positive impacts on the host, resulting in improved growth and health (79).

## Avian Gut Microbiome-Metabolic Activities

The poultry GIT is essentially coated in a dense layer of commensal bacteria in a diverse array of niches. Generally, the most complex microbial communities are found in the crop and the ceca. There is less colonization in the intestines based on the unfavorable environment. For example, the duodenum contains numerous enzymes, high concentrations of antimicrobial compounds, such as bile salts, and also has a rapidly changing environment due to reflux from the jejunum up to the gizzard (80). Traveling further down the GIT, the ileum and ceca become more favorable environments with fewer enzymes and antimicrobial compounds; this is reflected in the increased concentrations of commensal bacteria, around 10^9^ and 10^11^ bacteria/g, respectively (46). The unique anatomical structure of the cecum allows for the occupancy of fermentable substrates not widely available in different areas of the GIT; this enables differing microorganisms to reside and produce large amounts of energy metabolites to aid in achieving the bird’s energy requirements (81).

Research profiling whole body energy consumption patterns has attributed 22.8% to being utilized by the GIT and liver (82), but not all of that energy is actually being used by and for the host. It was reported that the presence of GIT microbiota significantly increased the dietary metabolizable energy in the broiler chicken host, indicating that the microbiota are responsible for utilizing the additional dietary energy (83). The commensal bacterial communities utilize nutrients from the host’s diet as energy sources, making those nutrients unavailable to the host. However, they are able to produce short-chain fatty acids (SCFAs) from the fermentation of those nutrients (84). Research suggests the GIT microbiota aid in digestion and energy release from starch and fibrous contents, especially in the ceca. It is proposed that the amounts and types of SCFAs that are generated in the ceca are in proportion to differing starches that enter the ceca (85). Although SCFAs serve as additional energy sources for the host, it is suggested that only a proportion (up to 25%) of the overall SCFA energy is recovered by the bird (85, 86). In high-fiber and low-energy diets, bacterial digestion of the fiber also releases energy in the form of SCFA (84, 87). Along with generating accessible energy, the gut microbiome is associated with conservation of energy when nutrient sources (proteins, fats, and sugars) are low (88, 89). The production and absorption of SCFAs in the intestine are occurring continuously, with more or less being produced due to alterations in the diet or cecal microbiome (85).

Conversely, the resident microbiome has also been associated with unfavorable effects to the host’s utilization of dietary energy. Although the presence of the GIT microbiota has indicated a significant increase in levels of metabolizable energy in conventionally raised broiler chickens when compared to germfree (89), the metabolizable energy is attributed to the products generated by the GIT microbiota. The variation can be associated with the digestibility of those energy sources (dietary fiber and starches) being broken down into monosaccharides and SCFA. The SCFA are portrayed as possessing a high metabolic energy value, yet they are inefficiently utilized by the host. Therefore the levels of SCFA present are not reflective of the net deposition of energy to the host (86, 89). Another potential explanation may be that the presence of the gut microbiota increases the cost of energy by altering the rate of energy-consuming reactions (89, 90). For example, pathogens attach to the epithelial lining, alter its integrity and function, and in turn stimulate the renewal of epithelial lining, which increases the amount of dietary energy spent on gut maintenance (27, 91). It has also been observed that conventionally raised birds have higher energy requirements for maintenance when compared to germfree birds (92). This may be due to the addition of the host’s microbiota usage of metabolizable energy, or the host’s microbiota making dietary energy unavailable to the host (92).

## Avian Microbiome and Foodborne Pathogens

The complex lining of the lower intestines with bacteria serves as a barrier against colonization of pathogenic bacteria, which if allowed to occur, could lead to infection. The bacteria that settle first in the lining of the intestines necessitate that any other microorganisms in search of new residence must compete for space and nutrients in order to survive and colonize (80, 93, 94). Establishing the early foundation of a mature GIT microbiota has been associated with prevention of infection with pathogens, namely *Salmonella*, by beneficial bacteria outcompeting the pathogenic bacteria for space and nutrients (95–98). In nature, chicks are hatched in the presence of maternal fecal contents, allowing rapid colonization of members from the maternal gut microbiome (25). In an attempt to colonize newly hatched chicks with a mature and healthy microbiome that will discourage pathogenic bacteria from colonizing, chicks have been experimentally inoculated with competitive exclusion culture mixtures (97, 99–102). Introduction of the competitive exclusion cultures has proven to be effective in protecting young chicks from enteric pathogens and several reviews have been written on various aspects of this research (103–106).

As previously mentioned, commensal bacteria produce SCFA, which are recognized as having growth-inhibiting effects on enterobacteriaceae (107–109). The presence of the SCFA causes a drop in cytoplasmic pH, which is recognized as a contributing factor to the inhibition of pathogen growth (110). Although the mechanisms of SCFAs are not well understood, they are known to exhibit bactericidal and bacteriostatic properties (30–32, 111). Russell (30) suggested that it is not only the result of a drop in pH caused by the SCFA but also the uncoupling reactions produced by the translocation of protons by SCFA that contribute to the growth inhibition effects seen. In accordance with this notion, Davidson et al. (112) suggested that because the fatty acids produced are weak acids, they are effective as antimicrobials in their undissociated forms as they are able to easily diffuse through the cytoplasmic membrane of the microorganism. The fatty acids dissociate into anions and protons once in the cytoplasm of the microorganism (maintained relatively neutral or slightly alkaline), in turn decreasing the pH and causing conformational changes of cytoplasmic proteins, enzymes, and nucleic acids. In an attempt to reestablish a neutral/slightly alkaline pH, microorganisms utilize ATP-dependent pump systems to transport the anions and protons outside of the cell. This is in accordance with findings of Cherrington et al. (113), where incubation of *Escherichia coli* with propionic and formic acids resulted in reduced rates of macromolecular synthesis initially, yet it partially regained synthesis rates after continued incubation.

Anion accumulation is suspected to be another factor in uncoupling reactions that attributes to growth inhibition of bacteria in the presence of SCFA. It is suggested that the accumulation of acid anions causes an uncoupling effect of the electron transport chain from oxidative respiration (via the passage of molecules in their dissociated and undissociated forms, transferring protons into the cell to dissipate the proton motive force) as well as a chaotropic effect (disrupting hydrogen bonding in water causing macromolecules in solution to lose stability) that are accountable for the increased hydrogen ion leakage into the cell. The cell is unable to excrete hydrogen ions rapidly enough, making it difficult for the cell to regain its neutral/slightly alkaline intracellular pH (30, 110, 114, 115). The intracellular increase in hydrogen is unable to counteract the accumulation of acid anions (116). Another inhibitor of bacterial growth by SCFA is the disruption of the membrane of a microorganism by means of permeabilization or intercalation, allowing for the release of macromolecules and the destabilization of the membrane (117, 118). However, there are instances of pathogenic bacteria acquiring resistance to SCFAs (32). For example, pre-incubation of *Salmonella* with high concentrations of SCFA at neutral pH resulted in an acid tolerance response and has also been demonstrated to be responsible for modulation of virulence gene expression and attachment/invasion of *in vitro* tissue culture cells (119–122).

While the production of fatty acids is inhibitory to invading bacteria, studies suggested that the fatty acids are inactive against the species that produced them (123). Smulders et al. (124) found that acid-producing bacteria are tolerant to acids and in turn, the acidic environments that they generate. Therefore, the influences of the SCFAs produced by autochthonous bacteria may provide protection against pathogenic bacteria – *Salmonella*, coliforms, and *Campylobacter* – intent on colonizing in the intestine while leaving commensal bacteria unscathed (125). However, little else has been reported on the effects of the fatty acids on the producing species.

## Key Players in the Gut Microbiota

In the past, the microorganisms colonizing the GIT were thought to be commensal, neither beneficial nor harmful to the host, as opposed to being mutualistic (37). However, numerous germfree experiments in various animal models have indicated the value of these indigenous microorganisms (126–128). There has been overwhelming data collected revealing the beneficial impacts on both host physiology as well as immunology (75, 129). Several studies have indicated that introducing a balance of beneficial microorganisms to the poultry microbiota improves body weight gain and feed conversion ratio as well as warding off common diseases in poultry, such as Newcastle disease and infectious bursal disease (130–132). However, in order to better promote strategies for increasing the presence of beneficial bacteria, those bacteria and their interacting counterparts must be identified.

Although being incredibly diverse, the most abundant microorganisms in the gut microbiota of poultry are primarily anaerobic (54). This is somewhat expected since there is little to no oxygen available as an electron acceptor in the lumen, although the concentration of oxygen is greater toward the epithelium, thus forcing bacteria to use fermentation to produce pockets of organic acids within the lumen (133). Moreover, Sun and O’Riordan (133) suggest that as a result of this environment, it is necessary to investigate SCFAs more in depth because bulk analysis does not reveal the true nature and spatial arrangement of these acids (which would further indicate the location and family of anaerobic bacteria). There is no consistent data available indicating the overall Gram status of poultry GIT microbiota. Investigation into the commensal bacteria present in an untreated chicken ceca has resulted in an array of bacterial communities (Gram-positive Y-branched, Gram-positive non-sporulating, Gram-negative) and may be attributed to the rearing conditions, chicken breed, diet, or even the cultivation and enumeration methods applied for bacterial characterization (125). Nevertheless, there are trends observed in available data investigating the microbial populations in broiler chickens grown in a conventional poultry flock and those raised under laboratory conditions (76, 134).

Lactobacilli and bifidobacteria are two of the more well-known beneficial bacteria, however, there are numerous others: *Bacillus*, *Enterococcus*, *E. coli*, *Lactococcus*, *Streptococcus* as well as undefined mixed cultures (Table 3) (23). These bacteria are indigenous to the GIT, occupy space, and consume nutrients along the intestinal tract, limiting the colonization of pathogenic bacteria. In addition to competing for space and nutrients, these bacteria have been recognized for exporting bacteriocins, which can target and kill invading pathogens (133). All of these microorganisms fit under the umbrella term probiotics. Like prebiotics, probiotics also have specified criteria and characteristics: (1) non-pathogenic and of host origin, (2) resistant to gastric pH and processing/storage, allowing them to persist in the intestinal tract, (3) able to adhere to epithelial and mucosal membranes, (4) modulate immune responses, and (5) produce inhibitory compounds (23). It is the complexity and broad diversity of the beneficial microorganisms that make up the microbiome and allow for a mature and healthy host (51, 52).

**Table 3:** **Suggested microorganisms for potential probiotic use based on various characteristics**.

Microorganism	Host	Site isolated	Rationale	Reference
*Enterococcus faecium*	Chicken	Intestines	Bacteriocin-producing ability	(135)
*Pediococcus pentosaceus*	Chicken	Intestines	Bacteriocin-producing ability	(135)
Mixed culture^a^	Chicken	Cecum	Inhibition ability of *Salmonella*	(99, 136–139)
*Lactobacillus reuteri*	Chicken	GIT	β-glucanase gene enhances growth and nutrient digestion	(140)
*Lactobacillus fermentum*	Chicken	GIT	Intestinal adherence, pathogen inhibition, tolerance to gastric enzymes	(141)
*Bifidobacterium longum*	Chicken	GIT	Anti-Campylobacter activity	(142)
*Streptococcus faecium*	Chicken	GIT	Impacts of body weight, feed conversion, carcass yield, *Salmonella* colonization	(143)
*Streptococcus bovis*	Cattle	Rumen	Inhibition ability of *Salmonella*	(144)

*^a^Mixed culture composed of 29 cecal bacterial strains that have shown to inhibit Salmonella colonization*.

Bacteria may be beneficial to the host by aiding in degradation of polysaccharides otherwise indigestible to the host. The monosaccharides produced can be subsequently broken down further into SCFAs and lactic acid (37). As previously mentioned, both lactobacilli and bifidobacteria are beneficial and indigenous to the human and chicken GIT (145). Lactobacilli are members of a group collectively referred to as lactic acid bacteria, which metabolize carbohydrates to produce lactic acid as the primary end product (146). Oligosaccharides are their main nutritional source, which is reflected in their residence in ecological niches rich in carbohydrate-containing substrates, most commonly plant material, spoiled or fermented foodstuffs, and mucosal membranes of humans and animals (147). Along with their broad range of habitats, lactobacilli are able to adapt to various conditions by altering their strictly fermentative metabolism accordingly; they may be obligately homofermentative, facultatively homofermentative, or obligately heterofermentative (148). Their fermentative status is based on the levels and proportions of end products they generate from fermentation of differing substrates (although other factors, such as chemical and physical environment, play a role in determining fermentative status). Obligately homofermentative indicates that their primary fermentation product is lactic acid (>85%) generated by fermenting hexoses (149). Facultatively homofermentative indicates that they are capable of fermenting hexoses and pentoses using different pathways to generate lactic acid (although under low substrate concentration and strictly anaerobic conditions, they are capable of producing acetic acid, ethanol, and formic acid). Obligately heterofermentative lactobacilli ferment hexoses generating equimolar amounts of lactic acid, CO_2_, and acetic acid (148–150). Although the end products produced are a fair indication of fermentative status, they are not the sole factor. These microorganisms are aerotolerant and acidophilic, allowing for the GIT to be an optimal residence (146, 151).

Bifidobacteria are another well-documented example of beneficial bacteria. They are often associated with lactic acid bacteria for their production of lactic acid, however, they are phylogenetically distinct. Bifidobacteria are Gram-positive, heterofermentative, and non-motile (152). Like lactobacilli, bifidobacteria digest oligosaccharides to use as carbon and energy sources, to produce lactic acid, acetic acid, ethanol, and formic acid (153). They are not exclusive to the utilization of dietary compounds, they can also digest carbohydrates produced by other members of the GIT (154). Additionally, they are capable of internalizing simple sugars remaining in the environment, thus preventing pathogenic bacteria from utilizing them as a nutrient source (155).

Both lactobacilli and bifidobacteria are known to be members of the intestinal microbiota in animals and humans; their presence is important for the maintenance of the GIT microbiota (156–158). Being that lactobacilli and bifidobacteria are autochthonous and dominant in the GIT, they can be utilized as a control method of pathogenic bacteria by competition, for example *Clostridium perfringens* (156). Lactobacilli and bifidobacteria possess characteristics that allow them to out-compete pathogenic bacteria. Various strains of lactobacilli adhere to intestinal epithelial-like cells and exhibit antimicrobial activity against bacteria typically found in the (human) GIT (157). A link between the lactobacillus strain’s pH tolerance and antimicrobial properties has been reported, both *in vitro* and *in vivo* (157).

Different species of lactobacilli and bifidobacteria produce various antimicrobial agents, which allow them to be inhibitory toward pathogenic bacteria. Many species of lactobacilli and bifidobacteria produce SCFA; the production of these acids causes a drop in intestinal pH. The lowered pH level extends the lag phase for sensitive microorganisms (124). The undissociated forms of these acids are able to penetrate the microbial cell and hinder metabolic functions (further information on the mechanisms of these acids was discussed in a previous section of the current review). Another end product generated from lactobacilli and bifidobacteria is CO_2_, which has demonstrated inhibition of microbial growth (149). The inhibitory mechanism of CO_2_ is unclear, although Eklund (159) was able to rule out the proposed mechanism of CO_2_ accumulation in the membrane of the microorganisms, physically interrupting the bacterial membrane. Growth of *E. coli*, *Bacillus subtilis*, *Pseudomonas aeruginosa*, and *Bacillus cereus* has been shown to be inhibited in the presence of CO_2_ at various concentrations (159). Succinic acid is produced by both lactobacilli and bifidobacteria, although at minimal levels (160, 161), and is associated with antibacterial activities in a multitude of environments (162, 163). Diacetyl is an end product of lactobacilli that exhibits antimicrobial effects. It is suggested that diacetyl is more effective in a lower pH (≤7) causing it to be lethal to Gram-negative bacteria and inhibitory of yeasts (164). Bacteriocins, produced by lactobacilli, may have a narrow or broad range of activity. Lindegren and Dobrogosz (149) have reviewed the various antimicrobial agents produced by lactic acid bacteria in more detail.

Overgrowth of any single type of bacteria can have unfavorable effects on the host. Lactobacilli are considered beneficial bacteria, however, antibiotic growth promoters that stimulate improved growth of broilers were also associated with heightened sensitivity of lactobacilli to those antibiotics (165). Although the host may benefit from the commensal bacteria competing with pathogenic bacteria, an overgrowth of commensal bacteria can be detrimental to the host by excessive uptake of nutrients making them unavailable to the host (166). Additionally, overgrowth of lactobacilli can impair host fat absorption by not allowing proper biotransformation – deconjugation and dehydroxylation – of bile acids (14). Overgrowth of bacteria can also lead to overproduction of fermentation end products to the detriment of the host. For example, overgrowth of *Streptococcus bovis*, a commensal lactic acid-producing bacteria can generate considerable acid production and a concomitant lowering of the surrounding environment pH. This sequence can be advantageous for competing against pathogens. Consequently, under *in vitro* incubation conditions in co-culture with *Salmonella typhirmurium* growth of *S. bovis* can behave as a probiotic and directly limit *Salmonella* growth as a function of carbon source and time of inoculation (144). However, when easily fermented carbohydrates are fed to ruminants, excessive *S. bovis* growth can occur in the rumen resulting in rapid lactic acid overproduction, subsequent lowering of the ruminal pH, and the eventual development of a harmful ruminal lactic acidosis condition in the animal (167). Therefore, even though *S. bovis* might be considered a gut commensal organism, and in some cases a probiotic candidate, it can also be associated with host clinical disease states, such as bacterial endocarditis and colon cancer in humans (144).

## Introduction and History of Prebiotics

The most widely accepted definition of prebiotics are non-digestible feed ingredients that are selectively fermented by beneficial bacteria in the lower GIT (capable of withstanding harsh conditions in the upper GIT) so as to provide energy to promote bacterial growth and metabolism in the colon which contributes to specific changes that lead to improved host health (22, 35, 168). Colonic food is a non-digestible ingredient that makes it past the upper GIT and into the colon, serving as a substrate for non-specific bacterial inhabitants, both beneficial and harmful (169, 170). Not all colonic foods are necessarily prebiotics; the rationale for designating a compound as a prebiotic or not depends upon whether beneficial bacteria alone are able to digest it. Some miscellaneous compounds that serve as colonic food, but do not fall into the category of prebiotics because of the non-specific targeting of microbiome bacteria include resistant starch, non-starch polysaccharides, non-digestible oligosaccharides, and yeast fermentation products (171). There have been numerous studies conducted and reviews written covering common prebiotics and their beneficial impacts; therefore they will not be discussed in detail here (Table 4) (35, 172–174).

**Table 4:** **Published reviews on the considerations of common prebiotics in various hosts**.

Prebiotic	Considerations	Host	Reference
Inulin-type	Structure overview	Not applicable	(172)
Short-chain carbohydrates	Gut function and health	Human	(175)
Inulin-type	Bifidogenic, resistant to digestion	Non-specific	(176)
Resistant starch	Production of SCFA, microbiome modulation, gut-associated immunomodulation	Human	(177)
Mannan-oligosaccharides	Modulation of gut microbiome	Poultry	(27)
Fructo-oligosaccharide, galacto-oligosaccharide, lactulose	Criteria for prebiotic classification	Human	(168)
Inulin-type, oligofructose	Quantification of inulin and oligofructose in Western diet	Human	(173)
Fructo-oligosaccharide	Bifidogenic, lack carcinogenic and toxic effects	Poultry, swine	(174)
Fructo-oligosaccharide, inulin-type	Selective to beneficial bacteria, prevent pathogen colonization	Poultry	(178)

Some lesser-studied prebiotic-like compounds are *Saccharomyces cerevisiae* fermentation products (SCFPs) or yeast culture (YC) components; these compounds do not fall into the precise definition of prebiotics as set by Roberfroid (22), among other classical definitions. However, they have prebiotic-like effects in that they have been shown to enhance nutrient utilization and digestibility, as well as improving the immune system and inhibiting pathogen-intestinal cell interaction by modifying the GIT microbiome (179–181). The fermentation of *S. cerevisiae –* undefined strains – produces SCFP. They include the fermentation products and metabolites, the media used in the fermentation to preserve fermentation activity, and both the yeast cell wall fragments and residual live yeast cells; thus, they share characteristics in both probiotic and prebiotic realms (179). There are commercial YC products available that are being more thoroughly investigated to identify their exact effects and maximize the directed influence(s) they may have.

Because yeasts are most often associated with the wine making, brewing, baking, and other fermenting industries, it is critical to consider why these unique organisms were initially promoted for use in improvements of animal and human health. In order to do this, a brief review of the history of yeast that led to its usage as a feed additive is discussed in the following section.

## Introduction to Yeast: History and Background

To understand the current use of yeast and yeast products in food and agricultural settings, it is important to at least briefly describe the history of yeast in scientific applications and the evidence for the close relationship among yeast strains originally uncovered and those used in today’s laboratory-based research. Humans began using yeast over 7,000 years ago, with its earliest usage dating back to the Neolithic times for wine making (182, 183). In the past century, yeasts have been investigated on a genetic level after the Carlsberg Laboratory introduced scientific concepts to the brewing industry, as discussed by Greig and Leu (184). In the 1930s, the genetic analysis of yeast became accepted based on its potential as an experimental organism; it was pioneered by Øjvind Winge and Carl Lindegren (185). Winge used a strain isolated from the Carlsberg Laboratory, while Lindegren used a strain, EM93, isolated from rotting Californian figs (182). Yeast continued gaining popularity in the scientific field for its ease in gene manipulation (182). In the 1950s, Robert Mortimer constructed the strain S288C, which has been purported to share more than 85% of its genome with EM93, Lindegren’s original strain (most laboratories involved in the analysis of yeast use a derivative of EM93 – a strain of *S. cerevisiae*). This strain was subsequently sequenced in 1996, making it the first fully sequenced yeast genome (186, 187). For further purposes of the current review, *S. cerevisiae* is the main species of yeast discussed unless otherwise indicated.

## Yeast in the Laboratory

A renowned model organism, yeast is a single-celled fungal eukaryote that most often divides by budding. Yeasts are used in various industries because of their ability to ferment sugars in the absence of oxygen to produce CO_2_ and alcohol. In a laboratory setting, yeasts are most often used for analysis as a model template to study higher eukaryotic organisms. Yeasts are ideal for studying processes known to occur in more complex eukaryotic organisms because even though yeasts are unicellular, they encode similar proteins and are thus representative of more complex organisms at the cellular level (188). When comparing all yeast protein sequences to mammalian sequences, of the potential protein encoding regions in yeast, “statistically robust” homology among the two was observed (189). Because of the lack of mammalian protein families and proteins sequenced, there may be much greater similarities between the two.

Part of the attraction of yeast as an experimental model is the ability to easily manipulate and mutate genes, either on plasmids or in the yeast chromosome itself, to view the resulting phenotypic effects (182). An insight into its fairly simple manipulation is evident in research performed by both Caspeta et al. (190) and Liu et al. (191). Caspeta et al. (190) manipulated *S. cerevisiae* into expressing thermotolerance to temperatures ≥34°C (typical response to these temperatures is serious impairment of function) by exposing the isolate for short stretches of time to increased heat followed by serial batch transfers. This resulted in non-inheritable heat tolerant strains that exhibited increased growth rates as well as increased glucose consumption rates at higher temperatures when compared to thermolabile strains (190). Thermotolerance has also been bestowed upon *S. cerevisiae* by the introduction of genes from organisms that are naturally thermotolerant. This transfer of genes allows for inheritable alteration in future generations of *S. cerevisiae*. Duina et al. (182) illustrated the extent to which yeast has proven its efficacy as a model organism, discussing research advancements and accolades (Nobel Prize and Lasker Award) in an array of fields achieved by utilizing yeast.

Although great progress has resulted from the study of yeast, it has also stimulated further inquiry. Yeast researchers began with the goal of determining functions of single genes and proteins, but now seek a “systems level” approach. The benefit of understanding how proteins interact to maintain cellular functions (metabolism, reproduction, growth, regulation, signaling, and homeostasis) is now at the forefront for yeast biology (192). Yeast’s position as a model organism for various scientific fields is reviewed more thoroughly in several articles and therefore will not be further discussed here (192–194). A review by Siddiqui et al. (195) encompasses the potential of engineering yeasts to contain secondary metabolite pathways for pharmacological purposes. Additionally, Sherman (196) has generated a comprehensive review (both extended and truncated versions available) on the biological basics of yeast, which includes a section on a variety of outside literature references for yeast.

## Yeast Metabolism

Yeasts are capable of cellular respiration in the presence and absence of oxygen; for this review, we will discuss respiration only in the absence of oxygen, as it is most applicable to the topic of the current review. Anaerobic respiration, or fermentation, is the process of breaking down sugars to generate energy for carrying out cellular processes. In anaerobic cellular respiration, sugars are broken down into pyruvate and subsequently decarboxylated and reduced to form CO_2_ and ethanol. For fermentation to begin, any complex sugars must be broken down into simple sugars (e.g., sucrose to glucose and fructose) via enzymes from yeasts, adding an additional step to the fermentation process (197). In the process of understanding this, it is recognized that complex carbohydrates (starches and fiber) are more challenging for yeasts to ferment than simple sugars. Investigation into the types of sugars and environments yeasts are capable of fermenting is necessary to optimize the production and utilization of yeast fermentation products. By understanding the conditions in which yeast fermentation is optimized, they can be engineered to generate additional metabolites that may prove to be beneficial for use in animal feed.

## Yeast as an Animal Feed Additive

The usage of live yeast and yeast products in animal feed is not a new concept, although pinpointing the exact point of its conception has proven to be challenging. It is suggested that the introduction of YC in animal feed was not until the 1980s (198). It appears that the majority of research has been dedicated toward ruminants, while equine, porcine, poultry, and companion animals received attention to a lesser extent. Initially, yeast was used in an array of modes because of the large quantities of yeast biomass waste generated by distilleries (and other yeast utilizing industries) (199). It was used as a feed additive because it was a rich source of protein, fiber, and minerals. It has been hypothesized that both viable and non-viable yeast cells provide essential B vitamins and organic acids (200). In the past, both viable and non-viable yeast cells have been added to animal feed – including poultry feed – and resulted in increased host growth and improved health (199).

It is essential to have a precise definition for YC, so it is not confused with using live yeast (probiotic/direct fed microbial form) or yeast extract (only soluble portion of yeast autolysis) products (201). As described in a previous section of the current review, YC contains the cellular constituents as well as residual viable cells. It is effective when used because it contains lysed yeast cells; this allows for the nutrients within the yeast cells to be available for digestion and absorption (202). These yeast cells are lysed by autolysis; they are subjected to temperature or osmotic shock, thereby killing the yeast cell while leaving the endogenous enzymes undamaged. The yeast cell’s own enzymes begin to degrade the yeast cell, releasing its contents and further degrading its proteins into amino acids (203). Some yeast cells that are capable of tolerating the temperature or osmotic shock, do not autolyze, and remain metabolically active.

The mode of action of YC is seen to enhance digestive and fermentative functions of the GIT, while modifying activities of the GIT microbiota, although the mechanisms are less clear (198). Based on *in vitro* and *in vivo* studies, supplemented YCs appear to have several impacts on the rumen microbiota including increased numbers of beneficial bacteria and fiber digesting bacteria as well as shifting away from hydrogen consuming methanogens and toward bacteria capable of converting hydrogen and CO_2_ to acetic acid, all of which could, in turn, potentially benefit the ruminant host animal either directly or indirectly (204, 205). Enhanced growth performance resulting from the supplementation of YC with probiotics (*Lactobacillus acidophilus* and *Streptococcus faecium*) has indicated its potential effect of increasing digestion and absorption of the GIT microbiota occurring in broiler chickens (206). de Oliva Neto et al. (207) conducted studies on the antibacterial properties of YC supernatant, which indicated a reduction of pathogenic bacterial growth when tested against a common distillery bacterial species. Interestingly, the supernatants were tested as both fresh and post freeze/thaw, and reported similar results indicating the antimicrobial activity could withstand freezing. Conversely, when heat (90°C for 20 min) was applied, the antibacterial activity was destroyed. Accordingly, YC and yeast extract have yielded varying results, which suggests the necessity for metabolically active yeast cells. When supplementing heat-treated inactive yeast cells to steer diets, there was no effect on the concentrations of cellulolytic bacteria, while supplementing live, metabolically active yeast cells increased the concentration of cellulolytic bacteria (208).

In addition to their ability to interfere with bacteria due to their relative large size, supplementation with live yeast products has led to a few suggested modes of action (209). One mechanism suggested by Jouany et al. (204) involves metabolic competition with bacteria that may be adhering to and digesting fiber or starch molecules. In this scenario, the yeasts ferment the carbohydrates produced, prohibiting their usage by other bacteria. Another mechanism of action of live yeast cells is their ability to produce protective products with antitoxin effects (210). Yeast intake has resulted in a stimulation of activity of host intestinal brush border enzymes, which has counteractive effects to those of pathogens, along with supplying the host with additional enzymes (211). Elimination of oxygen has been deemed the most influential mode of action in ruminants (212). Although there is little oxygen present in the GIT, live yeast cells scavenge for excess oxygen introduced by food and water intake; this allows for a more optimal environment for anaerobic bacteria (204, 212). Most all implications regarding the mechanism of oxygen elimination have been derived from studies conducted on ruminants.

As noted previously, the majority of the studies on the effects and mechanisms of YC have been performed on ruminants. Although such studies may be a good indicator of the potential use of YC in other animals, it can also be expected that there will be differences seen among ruminants and non-ruminants. For example, considerable research has been conducted on the effects of milk production in cattle, while this is beneficial for other lactating animals, the information gleaned from these studies holds little merit for poultry researchers. Instead, conducting *in vitro* and *in vivo* studies on specific animal subjects of interest would be more useful in identifying the mechanisms of YC in those animals rather than projecting ruminant/rumen microbiota results onto non-ruminant species.

## Impact of YC on Host–Microorganism Interactions

The effects of YC on the intestinal morphology in swine have indicated increased jejunal villi width, which allows for greater digestive and absorptive intestinal capacity leading to better body weight gain when compared to controls (180). In contrast, poultry data obtained has thus far indicated significant differences in intestinal morphology (213–215). Supplementation of YC has resulted in more shallow crypt depths, indicating less necessity for cell renewal and turn-over, allowing for decreased host energy utilization for intestinal epithelial maintenance (216). Feed efficiency and body weight gain have both resulted in significant increases when YC, yeast derivatives, and live yeast cells are added to the poultry diet (215, 217, 218).

Inclusion of YC in animal feed has led to suggestions that they may aid in the clearing of pathogens from infected animals. A study involving the inoculation of pigs with *Salmonella* suggested that the inclusion of YC in the diet allowed for rapid shedding of the pathogen from the GIT (180). Supplementation of broiler feed with YC has also been seen to enhance adaptive immune system T lymphocytes, allowing for better clearing of the pathogens (181). El-Husseiny et al. (219) observed that commercial YC were able to significantly increase antibody production against SRBC, much in agreement with the findings of Al-Homidan and Fahmy (220), who reported significantly higher antibody titer concentrations in response to Newcastle disease in broilers fed YC.

Further examination into the components of yeasts’ cell walls indicates the beneficial structural polysaccharides present and released into culture when yeast cells autolyze. Mannan-oligosaccharide (MOS) is included in the YC as it is derived from the outer cell wall of *S. cerevisiae*. MOSs bind to pathogenic bacteria in the GIT, preventing their attachment to the mannan residues on intestinal epithelia (221). This not only protects the host from pathogens but also allows for host energy reserves to be utilized for their own growth rather than to the repair and regeneration of the epithelial lining (222). β-glucans are also released when the yeast cell wall is degraded; presence of these molecules can lead to pathogen inhibition along with immuno-modulating effects. Similar to MOS, β-glucans act by preventing pathogens from binding to the villi of the gut mucosa (214, 216). Additionally, β-glucans are known to activate phagocytes, natural killer cells and B and T lymphocytes as well as increase cytokine production and phagocytic activity of macrophages (223).

Mannan-oligosaccharide supplementation has been reported to increase broiler growth performance when supplemented in their diet (224, 225). *In vitro* experimentation has indicated that addition of MOS inhibits the attachment of enteropathogenic *E. coli* to the gut mucosa as well as removing attached *E. coli* from the mucosa (226). Inclusion of yeast fermentation products, like MOS, appears to reduce pathogenic bacterial populations. The mechanism is unclear, although the agglutination of the pathogens with sugars from the yeast cell wall occurs rather than attachment to the host intestinal lining is one hypothesized mechanism (227). Yang et al. (228) indicated MOS altered the gut microbiota of broilers and reduced the number of mucosal-associated coliforms.

Although some studies suggest a positive association between yeast and growth promotion (229, 230), other studies have indicated no positive effects on inclusion of YC in broiler diets (231). Paryad and Mahmoudi (229) indicated that inclusion of 2% yeast (*Saccharomyces cerevisae*) in broiler chicken diets resulted in significant differences in body weight gain, feed intake, and feed conversion rate when compared to controls. Similarly, investigation into YC on growth promotion in lambs suggested its efficacy, resulting in increased feed intake and growth by 8 and 26%, respectively. Conversely, similar research conducted on lambs evaluating the efficacy of three yeast strains and a mixed culture resulted in little consistency and lacked an overall effect when compared among yeast strains (232). Adebiyi et al. (231) also showed no significant differences in body weight gain in broiler chickens when fed varying percentages of YC.

## Yeast Metabolites and Metabolism as Prebiotic-Like Substances

In addition to the structural polysaccharides derived from the yeast cell wall, yeasts generate a number of metabolites that may offer benefits to the host animal when supplemented to animal feed. Metabolites include carotenoids, vitamins, enzymes, amino acids, and some miscellaneous products (200). Several yeast species are naturally capable of producing carotenoids (including β-carotenes), which are subsequently metabolized into vitamin A (200). Vitamin A aids in cellular differentiation and proliferation, making it critical for intestinal maintenance and health (233). The enzyme responsible for the synthesis of vitamin A from β-carotene is β,β-carotene 15,15′-monooxygenase, which has been isolated and characterized from the intestines of poultry, among other animals (234, 235). Although *S. cerevisiae* is not capable of naturally producing carotenoids, it is capable of and has been engineered to express a biosynthetic pathway for the production of β-carotene (236).

Other vitamins (vitamin precursors) produced by yeasts include ergosterol, l-ascorbic acid, and d-erythroascorbic acid. Ergosterol is particularly abundant in *S. cerevisiae*, accounting for up to 90% of the total sterols (237). It is located in the membrane of yeasts and is responsible for its fluidity, structure, permeability, and activity of membrane-bound enzymes (238). Ergosterol is a precursor to both vitamin D_2_ and cortisone (239). Vitamin D_2_ is responsible for the proper absorption and transport of calcium, among other minerals (240). d-Erythroascorbic acid is also synthesized by *S. cerevisiae* and depending on the substrates available, that pathway can be manipulated into producing l-ascorbic acid (vitamin C) (241). The ingestion of vitamin C has been suggested to alleviate some of the repercussions of heat stress: poor immune function and growth performance (242). However, instances of supplementation of l-ascorbic acid in poultry diets have had varying results; some resulted in increased levels of superoxide dismutase in 45-week-old broilers, while others revealed no effect on the activities of antioxidative enzymes, superoxide dismutase included in 7-week-old broilers (243, 244).

Yeasts are recognized for their production of enzymes expressing various activities (245). Jones (246) wrote a comprehensive review documenting the activities of the proteolytic systems in *S. cerevisiae*, along with mentioning other enzymes elucidated in *S. cerevisiae* (carboxypeptidases, aminopeptidases, and dipeptidyl aminopeptidases). An enzyme in *Saccharomyces boulardii*, a subtype of *S. cerevisiae*, was found to degrade the ileal receptors in rats for toxin A generated from *Clostridium difficile* (a food-associated pathogen causing gastroenteritis; one study isolated *C. difficile* from 2.3% of broiler chickens tested) (247, 248). The degradation of the receptors prohibits the toxin from binding and prevents infection from occurring (249, 250). There have been multiple other proposed mechanisms of action for yeast on the immunoprotective effect in the GIT, specifically the prevention of *C. difficile* infection: (1) *S. boulardii* releases proteases that hydrolyze toxins and prevent its binding to the intestinal receptor (250), (2) *S. boulardii* is capable of stimulating the activity of disaccharidases in the intestinal brush border with no additional alterations of the intestinal mucosa (211), and (3) *S. boulardii* increased the production and secretion of glycoproteins, namely the secretory component of immunoglobulin A (251). Potentially, by narrowing the focus on the exact mechanism of action, *S. cerevisiae* could be engineered to confer said mechanism and supplemented into animal (poultry) feed to prevent colonization of *C. difficile*.

Invertase is another enzyme produced by *S. cerevisiae*; it hydrolyzes sucrose into glucose and fructose (252). Invertase efficiency and sucrose availability allows for glucose to be a carbon source for *S. cerevisiae* (252). Ideally, provided the diet contained appropriate levels of sucrose, one could engineer *S. cerevisiae* to overproduce invertase and subsequently add it to poultry feed. This would allow increased production of glucose, available not only for its own needs but also for other microorganisms in the surrounding environment. This mode of action would not be selective toward beneficial bacteria in the microbiome.

Yeasts have multiple amino acid transport systems; amino acids are incorporated into proteins or they are broken down and utilized as nitrogen and carbon sources to promote growth (253). Yeasts and yeast derivatives are capable of producing amino acids; therefore supplementation to animal feed would provide both the host and the microbiome with amino acids. Almquist (254) reviewed the essential amino acid requirements in young chicks, laying hens, and turkeys; Almquist included a table outlining the percentages of each amino acid to reach a specific protein level. Amino acids are necessary for poultry to have proper growth and promote efficient weight gain and feed conversion ratios (255). Lysine appears to be one such amino acid that plays a significant role in the body composition of poultry (256). Mutants of *S. cerevisiae* have been revealed to produce up to 17 times as much lysine as wildtype; thus this rich source of lysine may prove to be valuable to the growth and development of poultry (257).

Miscellaneous metabolites are also produced in *S. cerevisiae*, including toxins responsible for the “killer phenomenon.” Originally, this phenomenon was considered to be lethal only toward members of the same species; however, further investigation has led to the recognition of these toxic species to have destructive consequences reaching both prokaryotic and eukaryotic organisms (258–261). Polonelli and Morace (261) acknowledge that the inhibition of outside species may not be a direct impact on the toxins secreted, but more of a concerted effort from multiple metabolites. Nevertheless, these toxic species of *S. cerevisiae* are displaying lethality toward unrelated species. This can be utilized to the advantage of commercial poultry production, provided further research is conducted on characterizing whether this toxicity also occurs toward beneficial bacteria.

## Conclusions: Impact on Poultry Industry and Future Directions

In the search for a replacement to antibiotic growth promoters, the poultry broiler industry has two main objectives, a substance that (1) increases the growth of broiler chickens (body weight gain and feed conversion ratio) and (2) prevents the colonization of invading pathogens. Ideally, a single feed additive would prevent pathogen colonization while developing beneficial microbiota to aid in bird growth and feed conversion (262). Multiple feed additives have been attempted: antimicrobial agents, probiotics, prebiotics, and prebiotic-like substances. Probiotics need to be clearly identified and carefully analyzed to understand the influence they may have on the poultry GIT microbiota. As discussed previously, lactobacilli and bifidobacteria are two known groups that provide the host health and well-being based on their end products. These bacteria both ward off pathogens by creating an unfavorable environment against pathogen retention in the gut and also generally aid host GIT health, in turn resulting in enhanced bird growth (133).

To increase the efficacy of supplying probiotics to the host, the concept of synbiotics has been suggested. Synbiotics entail equipping the beneficial bacteria with substrates specific to their metabolic needs (23). Potentially, this allows for the greatest impact as it reduces the substrates taken by the probiotics from the host. Prebiotic-like substances are often times non-selective, therefore, combining a probiotic and a prebiotic-like substance does not fit into the synbiotic definition (263). Understanding the effects and specificity of probiotics, prebiotics, and prebiotic-like substances will allow for the best match of known commensal bacterial communities and substrates for a given host.

Yeast cells and YC products developed thus far have been extensively examined for their effects as supplements in animal feed. Numerous studies report the positive association with growth performance, immunostimulation, and microbiome modulation in animals and humans (209). In addition to being explored for their positive impacts as supplements in animal feed, yeasts and their derivatives have been investigated for their low risk and assurances of safety in their usage. Yeasts are cost efficient in both production and formulation (200). They do not have the ability to transfer genes they may acquire to pathogenic or commensal bacteria, or to the host. Yeasts are able to resist acquisition of antimicrobial resistance as well as not allowing for the transfer of such resistance (209). This also allows yeast to be safely used in parallel with antibacterial agents. Yeasts also have multiple mechanisms of action, allowing them to be productive in a range of environments (200).

A more thorough understanding of the microbiome can elucidate the mechanisms of prebiotics and prebiotic-like substances. The GIT microbiome is distinct and unique in its functionality relying on the presence of a definable, and potentially identifiable, microbial consortia. Understanding the influences of the members of the microbiome and also the microbiome as a single entity will allow for a more directed approach in the search of therapeutics and growth promoters. The GIT microbiome may be more appropriately considered as an additional organ; it has impact on host growth and development, and host health.

The limitations in previous research conducted have made future research necessary to resolve unanswered questions. It is imperative to define universal and standardized detection methodology to identify the bacterial communities present in the healthy, mature poultry microbiome. This would alleviate the issue of having varying results based on detection methods utilized. In addition, evaluating the currently suggested probiotic candidate organisms (Table 3) indicates the potential advantages of involving multiple potential probiotic bacterial and yeast strains to exhibit a concerted effort in maintaining GIT health. This would allow for the identification of potentially more uniform mixed probiotic cultures consisting of functionally well-defined individual bacterial members that when used to inoculate newly hatched chicks ensures more rapid development of a mature beneficial microbiome.

Further work with yeast, YC, and yeast extracts needs to be conducted on poultry. Much of the discussion in the current review was based on the results from yeast products applied to animals and humans but not poultry. To gain an accurate sense of the effects in poultry, such experimentation needs to be conducted in poultry (*in vitro* and *in vivo*). Additionally, many of the metabolites mentioned previously were investigated independent of yeast, YC, or yeast extract. It would be beneficial to assess the impact of metabolites and components from yeast individually as well as when combined. This would allow for the identification of beneficial metabolites and their respective individual and combined functional impacts on the corresponding host.

## Conflict of Interest Statement

The authors declare that the research was conducted in the absence of any commercial or financial relationships that could be construed as a potential conflict of interest.

## Funding

Funding is provided internally by Dr. Steven Ricke’s lab.
